# Intraoperative 3-D mapping of parathyroid adenoma using freehand SPECT

**DOI:** 10.1186/2191-219X-2-51

**Published:** 2012-09-27

**Authors:** Kambiz Rahbar, Mario Colombo-Benkmann, Christina Haane, Christian Wenning, Alexis Vrachimis, Matthias Weckesser, Otmar Schober

**Affiliations:** 1 Department of Nuclear Medicine, University Hospital Muenster, Albert-Schweitzer-Campus 1, Gebäude A1, Muenster, 48149, Germany; 2 Department of General and Visceral Surgery, University Hospital Muenster, Albert-Schweitzer-Campus 1, Gebäude W1, Muenster, 48149, Germany

**Keywords:** Intraoperative probes, Freehand SPECT, SPECT/CT, Intraoperative mapping

## Abstract

**Background:**

Freehand single photon emission computed tomography (fSPECT) is a three-dimensional (3-D) tomographic imaging modality based on data acquisition with a handheld detector that is moved freely, in contrast to conventional, gantry-mounted gamma camera systems. In this pilot study, we evaluated the feasibility of fSPECT for intraoperative 3-D mapping in patients with parathyroid adenomas.

**Methods:**

Three patients (range 30 to 45 years) diagnosed with hyperparathyroidism (one primary and two tertiary) underwent parathyroid scintigraphy with technetium-99m sestamibi (^99m^Tc-MIBI) to localize parathyroid adenomas. Two patients were referred with persistent hyperparathyroidism after conventional parathyroidectomy. In all three patients, a planar scintigraphy of the neck was performed 10 min after injection (p.i.) followed by SPECT/CT (Symbia T2, Siemens Healthcare) and a correlative ultrasound 2 h p.i. ^99m^Tc-MIBI scan was performed the day before surgery in two patients and at the same day in one patient. fSPECT images were acquired intraoperatively using declipse SPECT (SurgicEye^TM^).

**Results:**

A total of five parathyroid adenomas were successfully located with SPECT/CT. fSPECT allowed intraoperative detection of all adenomas, and successful parathyroidectomy was accomplished. Parathyroid hormone level decreased intraoperatively in all three patients, on average, by 79% (range 72% to 91%).

**Conclusion:**

In this preliminary study, we could demonstrate that intraoperative localization of parathyroid adenomas is feasible using the freehand SPECT technology, thus allowing an image-guided parathyroidectomy.

## Background

**Figure 1:**
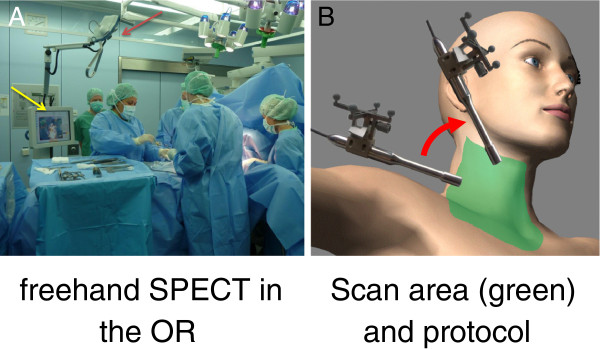
**Freehand SPECT in the OR and scan area (green) and protocol. **(**A**) Freehand SPECT device in the operating room. The device is placed next to the operating table with its stereostatic monitoring means (infrared cameras) looking at the operating field (red arrow). A touch screen enables user interaction and visualization of the reconstructed images (yellow arrow). (**B**) Scan area (in green) for a side of the neck including also the clavicular region. Scan protocol consists of painting this area with the gamma probe from the two different directions (red arrow) shown in the figure.

Technetium-99m sestamibi (^99m^Tc-MIBI) scan is the procedure of choice for the localization of parathyroid adenomas with improved accuracy using single photon emission computed tomography (SPECT) [1]. SPECT/CT has contributed to the localization of parathyroid adenomas by providing an anatomical context to scintigraphic images and correcting for attenuation effects [2]. Intraoperative localization using gamma probes has been proposed especially in minimally invasive surgery. This concept was the basis for the development of handheld imaging devices [3,4]. Freehand single photon emission computed tomography (fSPECT) was introduced lately as a three-dimensional (3-D) imaging and navigation technique designed for use in the operating room [5]. The technique is based on the use of a gamma probe, the position and orientation of which is stereotactically monitored while scanning an area of interest [5] (Figure 1A). ‘Scanning’ here means moving the gamma probe freely with the hand pointing at the body of the patient from different directions, i.e., ‘painting’ the surface of the patient with the gamma probe (Figure 1B). Each count rate acquired at a certain position can be seen as a one-pixel projection acquired by a one-pixel gamma camera (the gamma probe). A set of these one-pixel projections can be reconstructed into a 3-D image as in SPECT using also the information on its position and orientation. For visualization, the reconstructed images are then superimposed on a conventional video of the body surface, which is simultaneously recorded. The superimposition succeeds using augmented reality means, where virtual data (here, SPECT/CT or fSPECT images) are overlaid on the live video of an optical video camera. The use of intraoperative 3-D imaging for navigated extirpation of parathyroid adenomas might change the operation time and potentially the morbidity related to exploratory search of these adenomas using just the gamma probe. Furthermore, the possibility of controlling parathyroidectomy may reduce the need for reoperation. In this preliminary study, we apply the technique of fSPECT for the localization of parathyroid adenomas and evaluate its feasibility.

## Methods

A total of three consecutive patients (age range 30 to 45 years) undergoing conventional ^99m^Tc-MIBI whole-body scan to localize parathyroid adenomas before surgical procedure were additionally scanned intraoperatively using fSPECT. One patient suffered from primary hyperparathyroidism and two patients suffered from tertiary hyperparathyroidism. Preoperative SPECT/CT was performed routinely in all patients and served as reference imaging modality.

In all patients, a mean of 746 ± 12 MBq ^99m^Tc-MIBI was injected intravenously according to the German guideline for parathyroid scintigraphy. The injection of the radiopharmaceutical and the fSPECT were performed in the clinical setting. The data were analyzed retrospectively. Patients attending the University Hospital signed an informed consent for such anonymized data analysis. In one patient (#3), we used a 1-day protocol. To achieve a better lesion-to-background ratio, a 2-day protocol was performed in the other two patients. No re-injection before surgery was performed in all cases. Time to surgery was about 4 h in the 1-day protocol and about 24 h in the 2-day protocol.

Parathyroid hormone (PTH) levels were measured preoperatively before draping, followed by further samples at the time of extirpation and 10 min thereafter. fSPECT images were generated under sterile conditions shortly before the skin incision and when extirpating each parathyroid adenoma in order to control parathyroidectomy.

### SPECT/CT

The SPECT protocol consisted of 32 projections (180° using two opposing heads) of 10 s, each using a Symbia T2 hybrid scanner and low-energy high-resolution collimators (Siemens Healthcare, Erlangen, Germany). Low-dose CT was performed using a voltage of 130 kV with a tube current of 2.5 mA (20 mAs). SPECT/CT images were acquired with a reference marker to allow for navigation in the operating room.

### Freehand SPECT

The fSPECT protocol consisted of a 2- to 3-min scan (1,200 to 1,800 measurements, i.e., 20 measurements/second), covering one side of the neck and the corresponding periclavicular area using a declipse SPECT (SurgicEye, Munich, Germany) connected to a Crystal Probe (Crystal Photonics, Berlin, Germany) with a 40° collimator. The large number of measurements was achieved by continuously moving the probe. The device itself then decided which intervals are to be summarized as a complete projection. The scan was divided in a 1- to 2-min scan with the gamma probe pointing at a dorsal direction and a 1- to 2-min scan with the gamma probe pointing at a medial direction. Reconstruction was done using a modified ML-EM algorithm to take into account limited-angle acquisition. fSPECT images and preoperative SPECT/CT images were visualized as overlay on the live video of the patient or in a virtual reality view from the perspective of the tip of the gamma probe.

## Results

Patient's characteristics are given in Table 1. One patient suffered from primary hyperparathyroidism, and two patients suffered from tertiary hyperparathyroidism with a history of renal failure. ^99m^Tc-MIBI scan was performed the day before operation in two patients and at the same day in one patient. Parathormone level decreased intraoperatively by 79% (range 72% to 91%) in all three patients (Table 1). All adenomas (total of five, two patients with two adenomas each) were detected on whole-body scan, and SPECT/CT imaging helped to localize the adenomas precisely.

**Table 1:** Patient's characteristics

**Patients**	**Age (years)**	**Adenomas detected**	**Renal failure**	**Pre OP PTH (pg/ml)**	**Post OP PTH (pg/ml)**
1	30	1	+	906	81
2	45	2	−	202	43
3	33	2	+	1,149	328

PTH, parathormone; Pre OP, before removal; Post OP, after removal of adenomas; nomal values for PTH: 10 to 65 pg/ml.

**Figure 2:**
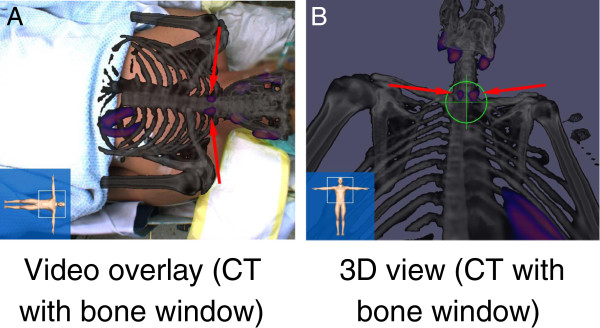
^**99m **^**Tc-MIBI SPECT/CT of patient 3. **Visualized as (**A**) video overlay on the live video of the patient and in a (**B**) 3-D virtual reality view of the SPECT/CT from the perspective of the gamma probe. The arrows show the parathyroid adenomas, and in (B), the target shows the direction the gamma probe is pointing at the time the screenshot was captured.

**Figure 3:**
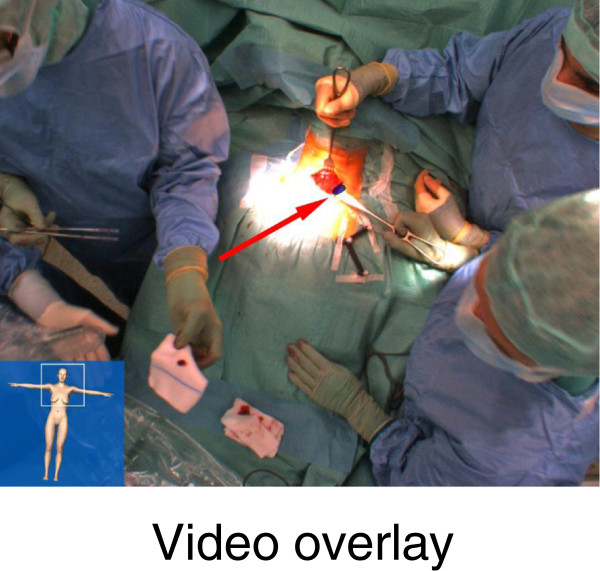
**Freehand SPECT of the left side of the neck of patient 2 as video overlay. **The arrow shows the adenoma in the image.

During surgery, SPECT/CT images (i.e., both the SPECT hotspots as well as the anatomy using different CT windows) were projected on the patient's body surface on the live video from the perspective of an optical camera placed above the operating table (‘video overlay’) as well as from the gamma probe in real-time (3-D view; Figure 2). Subsequently, fSPECT images (1,790 ± 647 measurements, 109 ± 62 s) were acquired for each side of the neck before preparation of that side, visualizing all five adenomas (Figure 2). Depth information provided by the system was used during preparation. After extirpation of each adenoma, freehand SPECT images were acquired to validate parathyroidectomy (Figure 3). No focal accumulation of radioactivity was found in the thyroid bed after surgery.

## Discussion

A total of five parathyroid adenomas were successfully located in SPECT/CT. SPECT/CT shows the lesion in the anatomical context, thus helping the experienced surgeon in finding the lesions. It is however not easy to mentally transfer transsectional images on the patient's body surface. fSPECT allowed precise intraoperative detection of all adenomas, and successful parathyroidectomy was accomplished. The clinical utility of fSPECT in the operating room still has to be evaluated in further studies.

The 3-D overlay visualization of the adenomas on the live video of the patient can help to reduce invasiveness of parathyroidectomy and also improve accuracy. However, to maintain accurate localization of parathyroid adenomas, which depends on exact intraoperative superimposition of images onto the operative field, the *situs* has to remain rather static. This can only be reliably accomplished by using a retractor system which warrants minimal changes of the operative anatomy during surgical manipulation. A higher impact is expected from localizing ectopic parathyroid adenomas in the mediastinum, but this has still to be evaluated.

**Figure 4:**
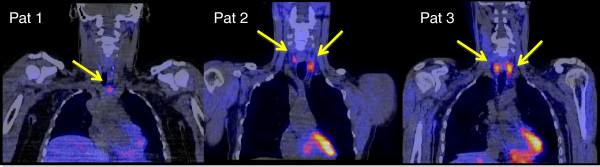
**Coronal slices of fused 3-D-reconstructed SPECT/CT images of all three patients. **Yellow arrows show the parathyroid adenomas in each image.

A new device like fSPECT causes not only costs in purchasing the device itself, but also daily costs for the fiducials. Hybrid imaging using SPECT/CT to localize parathyroid adenomas has high accuracy [6], and fused 3-D-reconstructed images can help surgeons in a similar way (Figure 4).

Since the scan must be repeated each time the patient is moved, several scans can extend the operation time.

## Conclusion

In this preliminary study, we could demonstrate that localization of parathyroid adenoma is feasible using freehand SPECT technology. This approach has high potential to allow image-guided extirpation of parathyroid adenomas, thus bringing imaging into the operation room. Larger comparative studies are needed to evaluate the additional value.

## Competing interests

The authors declare that they have no competing interests.

## Authors’ contributions

KR has made substantial contributions to the conception and design of the study and to the analysis and interpretation of data and drafted the manuscript. MCB and CH participated in the design and interpretation of the study and have critically revised the manuscript. AV and CW assisted in the design of the data analysis and revised the manuscript. OS and MW have conceived the study, participated in its interpretation, and have critically revised the manuscript. All authors read and approved the final manuscript.
